# Microenvironmental Alterations in Carbon Nanotube-Induced Lung Inflammation and Fibrosis

**DOI:** 10.3389/fcell.2020.00126

**Published:** 2020-02-28

**Authors:** Jie Dong

**Affiliations:** Receptor Biology Laboratory, Toxicology and Molecular Biology Branch, Health Effects Laboratory Division, National Institute for Occupational Safety and Health, Centers for Disease Control and Prevention, Morgantown, WV, United States

**Keywords:** inflammation, fibrosis, carbon nanotube, effector cell, soluble factor, extracellular matrix

## Abstract

Carbon nanotube (CNT)-induced pulmonary inflammation and fibrosis have been intensively observed and characterized in numerous animal studies in the past decade. Remarkably, CNT-induced fibrotic lesions highly resemble some human fibrotic lung diseases, such as IPF and pneumoconiosis, regarding disease development and pathological features. This notion leads to a serious concern over the health impact of CNTs in exposed human populations, considering the rapidly expanding production of CNT materials for diverse industrial and commercial applications, and meanwhile provides the rationale for exploring CNT-induced pathologic effects in the lung. Accumulating mechanistic understanding of CNT lung pathology at the systemic, cellular, and molecular levels has demonstrated the potential of using CNT-exposed animals as a new disease model for the studies on inflammation, fibrosis, and the interactions between these two disease states. Tissue microenvironment plays critical roles in maintaining homeostasis and physiological functions of organ systems. When aberrant microenvironment forms under intrinsic or extrinsic stimulation, tissue abnormality, organ dysfunction, and pathological outcomes are induced, resulting in disease development. In this article, the cellular and molecular alterations that are induced in tissue microenvironment and implicated in the initiation and progression of inflammation and fibrosis in CNT-exposed lungs, including effector cells, soluble mediators, and functional events exemplified by cell differentiation and extracellular matrix (ECM) modification, are summarized and discussed. This analysis would provide new insights into the mechanistic understanding of lung inflammation and fibrosis induced by CNTs, as well as the development of CNT-exposed animals as a new model for human lung diseases.

## Introduction

Pulmonary fibrosis is an irreversible pathologic stage that leads to high rates of morbidity and mortality in humans. It can result from a variety of human diseases, such as pneumonia, tuberculosis, and systemic sclerosis; develop spontaneously without a known cause, which is designated as IPF; be induced by a diversity of inhalational environmental fibrogenic irritants, such as silica dust and asbestos fiber, exemplified by human lung diseases silicosis and asbestosis; or be triggered by soluble chemicals, such as bleomycin and paraquat. Recently, a large number of studies have demonstrated that certain types of nanomaterials are potent fibrogenic agents in experimental animals. Despite the various causes, lung fibrosis is commonly featured by excessive deposition and accumulation of ECM materials, replacement of parenchymal tissue with connective tissue, and formation of permanent scars in lung interstitial space, which lead to destruction of alveolar and airway structures, stiffness of lung tissues, and loss of pulmonary function.

Carbon nanotubes (CNTs) are a class of nanomaterials with rapidly increasing annual production and a variety of industrial and commercial applications, such as electronic, energetic, and biomedical uses (De Volder et al., 2013; Zhang Q. et al., 2013; Abdalla et al., 2015). They are long and hollow nanostructures composed of either a single layer or concentric multiple layers of one-atom-thick carbon walls, which are designated as single-walled CNTs (SWCNTs) and multi-walled CNTs (MWCNTs), respectively. Most CNTs are respirable fibers with nano-scaled sizes, high aspect ratios, poor solubility, and substantial biopersistence. These physicochemical properties potentially associate CNTs to toxic fibers with fibrogenic activities, similar to asbestos (Donaldson et al., 2010; Johnston et al., 2010; Dong and Ma, 2015). Indeed, numerous studies performed in the past decade have demonstrated that certain types of CNTs behave as potent fibrogenic agents in the lung of exposed rodents. Meanwhile, CNT-induced lung fibrosis in animals possesses a high similarity to human IPF and pneumoconiosis (Dong and Ma, 2016b; Vietti et al., 2016). Owing to the increased exposure to CNTs and CNT-containing materials, the fibrogenicity of CNTs raises a serious concern over the adverse health impact of CNT exposure in human populations, which is supported by a few recent studies carried out in workers that were occupationally exposed to CNTs (Schulte et al., 2012; Fatkhutdinova et al., 2016; Vlaanderen et al., 2017).

Prompted by the noticeable fibrotic phenotypes triggered by CNTs in the lung, a large body of studies have been performed to elucidate the mechanisms that promote the initiation and progression of lung fibrosis in CNT-exposed animals in recent years, leading to a marked progress in this research area. Importantly, the findings reveal that the systemic, cellular, and molecular mechanisms of CNT-triggered lung fibrosis are consistent with the current knowledge on lung fibrosis derived from the studies on human fibrotic lung diseases and some lung fibrosis animal models, such as bleomycin-induced lung fibrosis, to a considerable extent, indicating CNT-induced lung fibrosis may serve as a new disease model (Dong and Ma, 2016b; Vietti et al., 2016; Dong and Ma, 2018b; Duke and Bonner, 2018). Remarkably, like the scenarios in many human fibrotic lung diseases, inflammation plays a critical role in the onset and progression of lung fibrosis induced by CNT exposure, providing the potential of using CNT-exposed rodents as a unique disease model for lung fibrosis studies (Dong and Ma, 2019b). To manifest the underpinning systemic, cellular, and molecular events that may function in the development of pathologic inflammation and fibrosis in CNT-exposed lungs, in this article, the major tissue microenvironmental alterations that are induced and function during the acute and chronic phase responses, including effector cells, soluble mediators, and functional events exemplified by immune cell polarization, fibroblast-to-myofibroblast differentiation, and ECM modification, are focused on for discussion.

## CNT-Induced Lung Inflammation and Fibrosis

Carbon nanotube-induced pulmonary pathological effects are characterized with biphasic inflammatory and fibrotic responses in experimental animals (Lam et al., 2004; Warheit et al., 2004; Muller et al., 2005; Shvedova et al., 2005, 2014; Mangum et al., 2006; Aiso et al., 2010; Porter et al., 2010, 2013; Park et al., 2011; Reddy et al., 2012; Mercer et al., 2013; Wang et al., 2013; Dong et al., 2015; Dong and Ma, 2016a, c, 2017a,b, 2018a). The lung injury initiates with a prominent acute inflammatory response, indicated by the rapid recruitment, infiltration, and accumulation of inflammatory cells in the interstitial, perivascular, and peribronchial regions in CNT-exposed lungs. During this stage, both type 1 and type 2 immune responses are activated. Briefly, type 1 response is marked by the differentiation and activation of Th1 lymphocytes and traditionally activated M1 macrophages, produces copious amounts of pro-inflammatory type 1 cytokines, such as TNF-α, IL-1β, and IL-6, and results in acute inflammation and tissue injury; whereas type 2 response is characterized by the formation and activation of Th2 lymphocytes that produce and release type 2 cytokines, such as IL-4 and IL-13, the polarization and activation of alternatively activated M2 macrophages stimulated by IL-4 and IL-13, the production of type 2 cytokines, chemokines, and mediators, such as TGF-β1, IL-10, CCL17, CCL18, and CCL22, and the functions in suppressing acute inflammation and promoting tissue repair and organ fibrosis. In CNT-exposed lungs, type 1 response erupts upon exposure and is predominant during the early acute phase, whereas type 2 response takes a longer time to occur and is dominant during the late acute phase. These events lead to strikingly elevated production and secretion of mediators, such as pro-inflammatory and pro-fibrotic cytokines, chemokines, and growth factors, which create a milieu that is capable of triggering and fostering fibrosis development. Concurrently with acute inflammation, a rapid-onset fibrotic response starts as early as day 1 post-exposure, which is demonstrated by elevated expression of fibrosis marker proteins, increased deposition of ECM proteins in alveolar septa, and enriched fibroblasts and myofibroblasts. With the persistent deposition of CNTs in lung tissues, the acute inflammatory and fibrotic responses reach an apex on day 7 post-exposure. After that the acute pathologic effects gradually decline, but transit to chronic responses. The chronic responses become fully established by day 28 post-exposure, last at least 1 year, and are featured by fibrosis and mild chronic inflammation. During the chronic phase, fibrosis is characterized with increased expression of fibrosis marker proteins, excessive deposition of ECM, accumulation of fibroblasts and myofibroblasts, thickened alveolar septa, and formation of fibrotic foci and epithelioid granulomas (Dong and Ma, 2016b; Vietti et al., 2016). The chronic immune outcome is dominated by type 2 response, in which alternatively activated M2 macrophages are enriched and activated (Dong and Ma, 2018b). In summary, the pulmonary responses to CNT exposure initiate from acute inflammatory and fibrotic events, and progress to chronic inflammation and fibrosis (Dong and Ma, 2019b). The biphasic process of CNT-induced lesions resembles that elicited by the deposition of fibrogenic foreign bodies, such as insoluble dusts and large biologic masses, in the lung. Moreover, pathologically, CNT-exerted pulmonary impact displays a considerable similarity to IPF and pneumoconiosis, especially when contemplating the observation that CNT-triggered lung fibrosis appears persistent and irreversible in animals.

A few animal models have been developed and studied for lung fibrosis in the past years, exemplified by bleomycin-, silica-, and irradiation-induced lung fibrosis. The advantages and disadvantages of these models have been reviewed previously (Moeller et al., 2008; Moore and Hogaboam, 2008; Degryse and Lawson, 2011; Williamson et al., 2015). Comparing with these models, CNT-induced lung fibrosis exhibits certain unique features. Remarkable acute immune response is induced in CNT-exposed lungs, which precedes and accompanies acute fibrotic response. This phenomenon is reminiscent of inflammation-driven fibrosis occurring in many human diseases, such as IPF, COPD, and systemic sclerosis (Dong and Ma, 2019b). CNT-exposed lungs therefore provide a model system to study the role of inflammation in the initiation and progression of fibrosis development, which cannot be performed in other fibrosis models due to the lack of marked acute immune response therein. CNT-induced lung inflammatory and fibrotic responses rapidly start within 1 day and persist for at least 1 year post-exposure, whereas bleomycin-induced lung fibrosis occurs 14–28 days post-exposure with spontaneous resolution thereafter, silica-induced lung fibrosis takes 12–16 weeks to develop, and irradiation-induced lung fibrosis may take more than 30 weeks to develop (Moore and Hogaboam, 2008; Dong and Ma, 2016b). Thus, CNT-exposed animals offer a system to study both the acute and chronic responses in inflammation and fibrosis, as well as the interactions between immune responses and fibrosis during each stage, which would enhance the understanding of relevant human diseases. Moreover, alveolar epithelial cell death (apoptosis) is remarkably induced and serves as a major trigger in bleomycin- and asbestos-induced lung fibrosis (Liu et al., 2013; Williamson et al., 2015). However, similar to the pathologic feature in IPF, alveolar epithelial cell death does not occur dominantly in CNT-exposed lungs. This finding indicates that the mechanism activated by CNTs to induce lung fibrosis differs from that by bleomycin or asbestos. Together, these observations demonstrate that CNT-exposed animals possess some distinct features to serve as a disease model for obtaining new mechanistic understanding of lung inflammation and fibrosis.

## Immune Cells and Soluble Mediators

**FIGURE 1:**
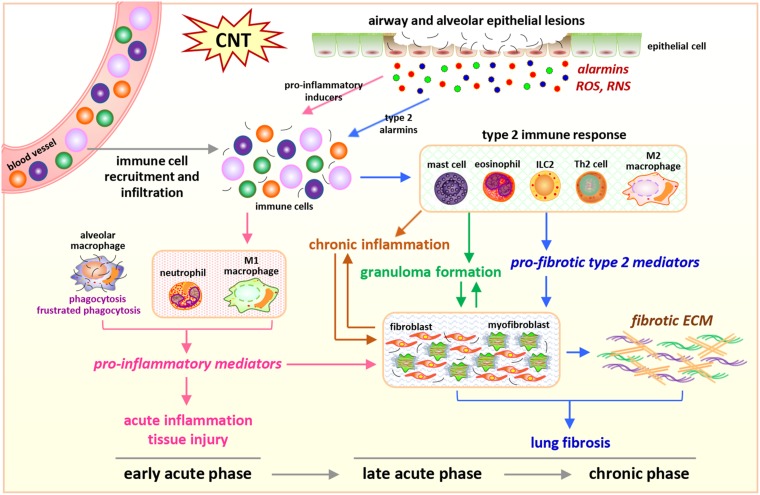
Integrated perspective of microenvironmental mechanisms in CNT-exposed rodent lungs. Enriched numbers of neutrophils and M1 macrophages and elevated levels of pro-inflammatory factors are hallmarks of acute inflammation induced by CNTs during early acute phase response. In contrast, promoted by certain alarmins produced by injured epithelial cells, Th2-driven type 2 immune response and fibrotic response are predominant during late acute phase response and chronic phase response, marked by increased type 2 immune cells, type 2 mediators, fibroblastic cells, and ECM production and remodeling in the lung exposed to CNTs. These induced cells, mediators, and events underlie the initiation and progression of CNT-induced inflammation and fibrosis in the lung.

In the lung exposed to CNTs, increased immune cells are observed over the entire course of inflammation and fibrosis, but with distinct subsets at different stages, which is in agreement with the cell type-specific functions of immune cells during disease development (Figure 1).

### Acute Inflammation

The hallmark of acute inflammation is the immediately and strikingly induced recruitment, infiltration, and accumulation of inflammatory cells (Dong and Ma, 2016b,2019b). Neutrophils and macrophages are markedly recruited and infiltrate into lung tissues, examined by cell counting of BAL samples and immunostaining of specific cell markers on lung tissues, which indicates their critical roles as the frontline responders in the onset of acute inflammation induced by CNTs (Shvedova et al., 2005; Porter et al., 2013; Dong et al., 2015; Rydman et al., 2015; Nikota et al., 2017; Dong and Ma, 2018a). At this stage, M1 (traditionally activated) macrophages are the dominant macrophage population, as determined by the cell surface markers CD86 and MHC II. A markedly increased number of macrophages express iNOS, a functional marker of M1, demonstrating the activation and function of M1 in the acute inflammatory response to CNTs. The activation of M1 is further supported by the increased levels of phosphorylated STAT1 and IRF5, two markers of M1-specific signaling pathways in macrophages. These findings reveal the pro-inflammatory and cytotoxic M1 functions in CNT-exposed lungs (Dong and Ma, 2018a). Meanwhile, increased lymphocytes are observed in BAL samples from CNT-exposed lungs, suggesting their roles in the consequent pathological responses (Shvedova et al., 2005; Dong et al., 2015).

The enrichment of inflammatory cells leads to markedly elevated production and secretion of pro-inflammatory cytokines and chemokines in CNT-exposed lungs. Functionally, cytokines are small secreted proteins, which bind to their specific receptors on target cells and activate downstream signaling pathways, leading to the transactivation of the genes that encode functional proteins; and chemokines are a family of small heparin-binding cytokines that are chemotactic and function in trafficking and orchestrating immune cells. A subset of cytokines and chemokines have been shown to exert pro-fibrotic functions as microenvironmental cues, leading to the initiation and progression of tissue fibrosis, in both animal and human lung fibrosis (Borthwick et al., 2013; Sahin and Wasmuth, 2013). For instance, the cytokines TNF-α, IL-1α, IL-1β, and IL-6, and the chemokines CCL2 (MCP-1), CCL5 (RANTES), and CXCL2, have been shown to play pro-fibrotic roles in bleomycin-induced lung fibrosis in mice and in certain human fibrotic lung diseases, such as IPF and asbestosis. Notably, one of the most predominant pathologic effects induced by CNTs is that a majority of these pro-inflammatory mediators are highly induced in CNT-exposed mouse, rat, and human lungs. The representative studies are listed in Table 1. These mediators may play important roles in the initiation and promotion of CNT-induced inflammation and fibrosis. The detailed analysis of their individual functions is emerging at the current stage. For instance, IL-1 signaling activated by IL-1α or IL-1β has been shown to promote acute inflammation, but not chronic inflammation or fibrosis, in the lung exposed to MWNT-7 MWCNTs, examined by using IL-1R KO mice (Rydman et al., 2015; Nikota et al., 2017). Insightful studies in this direction would identify the critical mediators among these rapidly induced factors to serve as functional biomarkers and therapeutic targets for lung inflammation and fibrosis.

**TABLE 1:** CNT-induced pathological factors in rodent lungs.

Group	Molecule	CNT	Animal	Sample	Method	References
Pro-inflammatory cytokine and chemokine	TNF-α	SWCNT, MWCNT	Mouse, rat	BAL	ELISA, MIA	Muller et al., 2005; Shvedova et al., 2005; Park et al., 2009; Han et al., 2010; Taylor et al., 2014; Dong et al., 2015; Frank et al., 2015
	IL-1α	MWCNT	Mouse	BAL	ELISA, MIA	Dong et al., 2015; Nikota et al., 2017
	IL-1β	SWCNT, MWCNT	Mouse	BAL	ELISA	Shvedova et al., 2005; Han et al., 2010; Taylor et al., 2014; Dong et al., 2015; Sun et al., 2015; Nikota et al., 2017
	IL-6	SWCNT, MWCNT	Mouse	BAL	ELISA, MIA	Park et al., 2009; Beamer et al., 2013; Taylor et al., 2014; Dong et al., 2015; Frank et al., 2015; Khaliullin et al., 2017; Nikota et al., 2017
	IL-12	SWCNT, MWCNT	Mouse	BAL	ELISA, MIA	Park et al., 2009, 2011; Nikota et al., 2017
	IFN-γ	SWCNT, MWCNT	Mouse	BAL	ELISA	Park et al., 2009, 2011
	CCL2	SWCNT, MWCNT	Mouse	BAL	ELISA, MIA	Dong et al., 2015; Khaliullin et al., 2017; Nikota et al., 2017
	CCL3	SWCNT	Rat	BAL	MIA	Fujita et al., 2015a
	CXCL1	MWCNT	Mouse	BAL	MIA	Rydman et al., 2015; Nikota et al., 2017
	CXCL9	MWCNT	Mouse	BAL	MIA	Rydman et al., 2015

Type 2 cytokine and mediator	IL-4	SWCNT, MWCNT	Mouse	BAL, tissue	ELISA, IHC, IF	Park et al., 2009, 2011; Dong and Ma, 2016a
	IL-13	SWCNT, MWCNT	Mouse	BAL, tissue	ELISA, IHC, IF	Park et al., 2011; Dong and Ma, 2016a
	IL-5	SWCNT, MWCNT	Mouse	BAL	ELISA, MIA	Park et al., 2009, 2011; Beamer et al., 2013; Nikota et al., 2017
	TGF-β1	SWCNT, MWCNT	Mouse, rat	BAL, tissue	ELISA, IHC, IB	Shvedova et al., 2005; Park et al., 2011; Dong et al., 2015; Sun et al., 2015; Dong and Ma, 2017a; Khaliullin et al., 2017; Nikota et al., 2017
	PDGF-A	MWCNT	Mouse, rat	BAL, tissue	ELISA, IHC	Ryman-Rasmussen et al., 2009; Cesta et al., 2010; Li et al., 2013; Dong et al., 2015
	IL-10	SWCNT, MWCNT	Mouse	BAL	ELISA	Park et al., 2009, 2011
	CCL11	MWCNT	Mouse	BAL, tissue	ELISA, IHC	Beamer et al., 2013; Dong and Ma, 2016a
	CHIA	MWCNT	Mouse	Tissue	IHC, IB	Dong and Ma, 2016a
	TIMP1	MWCNT	Mouse	BAL, tissue	ELISA, IHC, IF	Dong and Ma, 2017b
	OPN	SWCNT, MWCNT	Mouse, rat	BAL, tissue	ELISA, IHC, IF, IB	Huizar et al., 2011; Taylor et al., 2014; Fujita et al., 2015b; Dong and Ma, 2017a; Nikota et al., 2017
	MMP12	SWCNT	Mouse, rat	Tissue	IHC	Hsieh et al., 2012; Fujita et al., 2015b

ECM protein	Collagens	SWCNT, MWCNT	Mouse, rat	Tissue	MTS, PSRS, SSCA, HYPA	Muller et al., 2005; Shvedova et al., 2008; Chang et al., 2012; Mercer et al., 2013; Wang et al., 2013; Shvedova et al., 2014; Dong et al., 2015; Dong and Ma, 2016c
	Collagen I	SWCNT, MWCNT	Mouse	Tissue	IHC, IF	Zhang Y. et al., 2013; Dong et al., 2015; Dong and Ma, 2016c,2017a,b
	Collagen III	SWCNT, MWCNT	Mouse, rat	Tissue	IHC	Wang et al., 2013; Zhang Y. et al., 2013
	Fibronectin	MWCNT	Mouse	Tissue	IHC, IF, IB	Dong et al., 2015; Dong and Ma, 2017a, b

Alarmin	IL-25	MWCNT	Mouse	BAL	ELISA	Ronzani et al., 2014
	IL-33	MWCNT	Mouse	BAL, tissue	ELISA, IHC	Wang et al., 2011; Katwa et al., 2012; Beamer et al., 2013; Sager et al., 2014
	TSLP	MWCNT	Mouse	BAL	ELISA	Ronzani et al., 2014
	HMGB1	SWCNT, MWCNT	Mouse	BAL	ELISA	Jessop and Holian, 2015; Cui et al., 2019

*ELISA, enzyme-linked immunosorbent assay; IB, immunoblotting; IF, immunofluorescence; IHC, immunohistochemistry; HYPA, hydroxyproline assay; MIA, multiplex immunoassay; MTS, Masson’s Trichrome staining; PSRS, Picro-Sirius Red staining; SSCA, Sircol soluble collagen assay.*

### Type 2 Immune Response

In the late acute phase, neutrophils diminish in lung tissues, whereas macrophages persist (Shvedova et al., 2005; Dong et al., 2015; Rydman et al., 2015). T and B lymphocytes are enriched in the interstitial, perivascular, and peribronchial regions of the lung exposed to CNTs (Dong and Ma, 2016c). Th2 (IL-4+/IL-13+ CD4+) lymphocytes with increased levels of phosphorylated STAT6 and GATA-3 are induced in CNT-exposed lungs, demonstrating the differentiation and activation of Th2 cells by CNTs (Dong and Ma, 2016a). A number of studies have revealed that the levels of Th2-type cytokines, such as IL-4, IL-13, and IL-5, are significantly increased in BAL fluid and lung tissues of CNT-exposed mice, further supporting the activation of Th2 cells induced by CNT exposure (Dong and Ma, 2018b). IL-4 and IL-13 function as pro-fibrotic cytokines in driving fibrosis development in a variety of fibrotic diseases and animal models, indicating their potential roles in promoting CNT-induced lung fibrosis (Wynn, 2008; Wynn and Ramalingam, 2012; Borthwick et al., 2013; Wynn, 2015).

Correspondingly, in macrophage population, M1 cells decline, whereas M2 (alternatively activated) macrophages become predominant, as demonstrated by the M2 surface markers CD206 and CD163. The elevated number of ARG1 positive macrophages exhibits the functional activation of M2 at this stage. The M2 activation is also supported by the increased levels of phosphorylated STAT3, phosphorylated STAT6, and IRF4, which are the markers of M2-specific signaling pathways in macrophages, and the induced expression of two additional M2 marker proteins, FIZZ1 and YM1, in the lung (Dong and Ma, 2018a). M2 macrophages are known to produce excessive amounts of pro-fibrotic mediators, which can promote fibroblast accumulation, fibroblast-to-myofibroblast differentiation, and ECM production and deposition (Wynn and Barron, 2010; Murray and Wynn, 2011; Wynn and Vannella, 2016; Vannella and Wynn, 2017). Indeed, the levels of some of these mediators, including TGF-β1, PDGF, TIMP1, OPN, and MMP12, are significantly elevated in CNT-exposed lungs; meanwhile, TGF-β1, TIMP1, and OPN have been demonstrated to play essential pro-fibrotic roles in CNT-induced lung fibrosis (Dong and Ma, 2016b,2017a,b, 2018b). These findings reveal that M2 macrophages and their secreted factors are critical elements in the development of lung fibrosis induced by CNTs (Table 1).

Additionally, a few other type 2 immune cells play essential roles in the onset and promotion of type 2 immune response and fibrosis induced by CNTs in the lung (Dong and Ma, 2018b). For instance, MWCNT-triggered lung inflammation, fibrosis, and injury are markedly attenuated in mast cell-deficient *Kit*^*W–sh*^ mice and in ST2 (receptor for IL-33) KO mice that are defective in mast cell activation, indicating the critical role of mast cells (Katwa et al., 2012). Eosinophils are increased in BAL from MWCNT-exposed mouse lungs, accompanied by elevated levels of type 2 cytokines (Beamer et al., 2013; Rydman et al., 2014, 2015). ILCs are also observed to increase in MWCNT-exposed lungs (Beamer et al., 2013). These three types of cells are believed to function in producing the initial type 2 cytokines IL-4 and IL-13, which stimulate the differentiation and activation of Th2 cells leading to type 2 immune response, thus are of importance in CNT-induced lung inflammation and fibrosis.

The Th2-driven type 2 immune response can be activated by injury to suppress acute inflammation and promote tissue repair. However, when the insult and the injury caused are persistent or repeated, the activation of type 2 response and its repair function become prolonged and exaggerated, leading to pathologic organ fibrosis (Wynn, 2015; Gieseck et al., 2018). Accumulating evidence indicates that the exposure to CNTs and the induced activity of type 2 response fall into this scenario (Dong and Ma, 2018b). Thus, type 2 immune response plays critical roles in the transition from acute inflammation to chronic inflammation and fibrosis, and type 2 cytokines and mediators function as important microenvironmental cues, in CNT-exposed lungs.

### Chronic Inflammation

In the chronic response to CNT exposure, chronic inflammation with granulomas displays in the lung, accompanying fibrosis. At this stage, increased macrophages are present in BAL and lung tissues from CNT-exposed mice (Shvedova et al., 2005; Huizar et al., 2011; Rydman et al., 2015; Dong and Ma, 2017a). CD4+ T cells are induced dose-dependently in lung tissues on day 28 post-exposure to MWCNTs (Rydman et al., 2015). CD3+ T cells are enriched in granulomatous foci on day 60 and day 90 post-exposure to MWCNTs in mouse lungs (Huizar et al., 2011). Some pro-inflammatory cytokines exist at higher levels in CNT-exposed lungs than control lungs at this stage, indicating the occurrence of chronic inflammation (Table 1). The levels of certain type 2 mediators, such as TGF-β1 and OPN, in macrophages, BAL fluid, and lung tissues are remarkably higher in MWCNT-exposed lungs than those in control lungs during the chronic stage, suggesting the activation of type 2 immune response (Dong and Ma, 2018b). The presence of these immune cells and factors is consistent with their orchestrated roles in maintaining chronic inflammation and fibrosis, which persist for at least 1 year in CNT-exposed lungs. Nevertheless, compared with acute inflammation, the activities and mechanisms of chronic inflammation induced by CNT exposure are currently less studied and require further elucidation.

## Fibroblastic Cells and ECM Production

Studies on the mechanisms underlying pathologic fibrosis development have identified fibroblastic cells, i.e., fibroblasts and myofibroblasts, as major effector cells responsible for fibrosis, owing to their direct functions in excessive production of ECM components and pathological remodeling of injured tissues (Kuhn and McDonald, 1991; Zhang et al., 1994; Tomasek et al., 2002; White et al., 2003; Thannickal et al., 2004; Hinz et al., 2007; Wynn, 2008; Hinz, 2010; Wynn and Ramalingam, 2012). During fibrogenesis, tissue resident fibroblasts are regulated through stimulated migration, augmented proliferation, and defective apoptosis, leading to the accumulation of fibroblasts. Fibroblasts can be activated and differentiate into myofibroblasts, which possess contractile properties similar to those of smooth muscle cells due to *de novo* expression of smooth muscle proteins, such as α-SMA, and express exaggerated levels of ECM proteins exemplified by collagens and fibronectin, cytokines, and growth factors. Fibroblasts, myofibroblasts, and the copious ECM produced by them form fibrotic foci, which result in tissue scarring and organ dysfunction.

**FIGURE 2:**
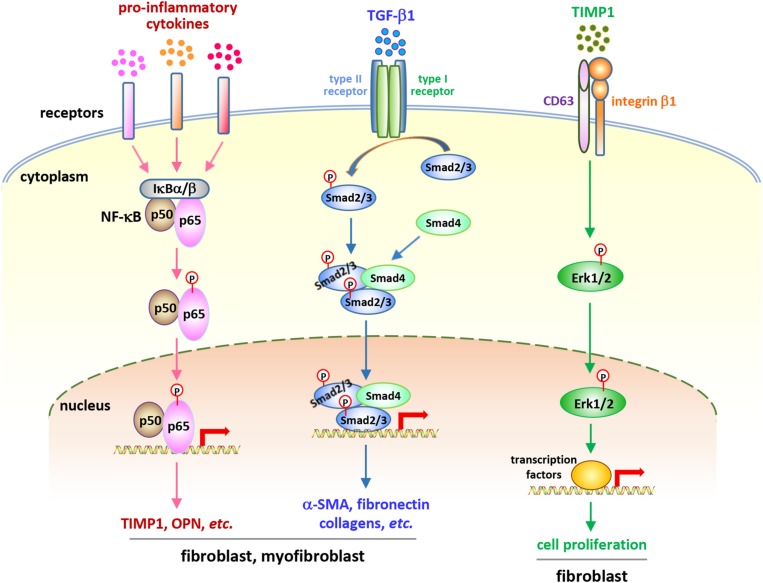
Soluble factor-activated signaling pathways in fibroblastic cells in CNT-exposed mouse lungs. Certain pro-inflammatory and pro-fibrotic mediator-regulated signaling pathways, including the canonical NF-κB pathway, the canonical, Smad-dependent TGF-β pathway, and the ERK signaling, have been reported to be activated in fibroblastic cells in CNT-exposed lungs. These pathways exert crucial functions in promoting the development of fibrosis induced by CNTs. They demonstrate the activities and roles of fibroblasts and myofibroblasts, as well as the connections between immune factors and fibrosis development, in the lung under CNT exposure.

Considering the central roles of fibroblasts and myofibroblasts in fibrosis development, studies on the behaviors and functions of these cells in CNT-exposed lungs have been performed in recent years. A number of studies demonstrate that fibrogenic SWCNTs and MWCNTs induce the enrichment of fibroblasts and myofibroblasts during both the acute and chronic phase responses in the lung, which have been summarized and discussed previously (Dong and Ma, 2016b). A few recent studies reveal that fibroblasts and myofibroblasts are activated and function in lung fibrosis triggered by CNT exposure. Meanwhile, emerging findings demonstrate that certain soluble factors generated from immune responses may function as the stimulators to activate critical signaling pathways in these fibroblastic cells, confirming the roles of, and the interactions between, immune cells and fibroblastic cells in the development of lung fibrosis induced by CNT exposure (Figure 2).

Comparison between WT and Opn KO mice reveals that OPN enhances MWCNT-induced lung fibrosis through promoting the formation of fibrotic foci and increasing the production of matrix proteins in the lung. At the cellular and molecular levels, OPN promotes TGF-β1 expression and activation, Smad-dependent TGF-β signaling activation and ECM protein expression in fibroblasts and myofibroblasts, fibroblast accumulation, myofibroblast differentiation, and ECM deposition in MWCNT-exposed lungs. By using TGF-β1 neutralizing antibodies and a type I TGF-β receptor inhibitor, it is confirmed that OPN enhances MWCNT-induced fibrotic response through activating Smad-dependent TGF-β signaling and elevating ECM production in fibroblastic cells. Together, these findings demonstrate that the induction of OPN and TGF-β1, and the activation and function of Smad-dependent TGF-β signaling in fibroblastic cells, promote MWCNT-induced lung fibrosis (Dong and Ma, 2017a). In another study, on day 7 post-exposure to SWCNTs, the induction of TGF-β1 protein is undetectable in the BAL from Opn KO lungs, whereas there is a 2.3-fold induction in the BAL from WT lungs; correspondingly, a decreased collagen deposition in Opn KO lungs on day 28 post-exposure is observed, compared with WT lungs (Khaliullin et al., 2017). These two studies establish TGF-β1 as a signaling mediator that associates type 2 immune response with fibrosis development in CNT-exposed mouse lungs.

Exposure to MWCNTs (MWNT-7) significantly increases the expression of the cell proliferation markers Ki-67 and PCNA and a panel of cell cycle-controlling genes in the lung in a TIMP1-dependent manner, determined by comparing WT mice with Timp1 KO mice. Accompanying the induction of TIMP1, MWCNTs increase the levels of CD63 and integrin β1 in fibroblasts and induce the formation of a TIMP1/CD63/integrin β1 complex on the surface of fibroblasts to trigger the phosphorylation and activation of Erk1/2, which might underlie MWCNT-stimulated, TIMP1-mediated fibroblast proliferation in the lung. Deficiency of TIMP1 in mice causes a remarkable attenuation in fibroblast enrichment, myofibroblast differentiation, fibrotic focus formation, and ECM deposition in the lung exposed to MWCNTs (Dong and Ma, 2017b). This study therefore reveals that TIMP1 is a pro-fibrotic factor in CNT-induced lung fibrosis in mice.

The pro-inflammatory cytokines, such as TNF-α, IL-1β, and IL-6, are well-known target genes, and in turn activators, of NF-κB signaling (Pahl, 1999; Ghosh and Karin, 2002; Lawrence, 2009). Enhanced or prolonged NF-κB activation has been detected in multiple lung diseases, such as asthma, COPD, and silicosis (Wright and Christman, 2003; Di Giuseppe et al., 2009; Edwards et al., 2009). The increased levels of pro-inflammatory cytokines in CNT-exposed lungs suggest the potential activation and roles of NF-κB in promoting the pathological effects. Indeed, two types of fibrogenic MWCNTs, MWNT-7 MWCNTs and long MWCNTs, markedly induce the activation of NF-κB signaling in fibroblasts and myofibroblasts in mouse lungs during both the acute and chronic responses, demonstrated by nuclear translocation of NF-κB subunit p65 and phosphorylation of NF-κB p65 Serine 276 (S276). Coincidently, two NF-κB-regulated genes encoding pro-fibrotic mediators, TIMP1 and OPN, are evidently induced in fibroblasts and myofibroblasts in MWCNT-exposed lungs, which confirms the transactivation of NF-κB and indicates the pro-fibrotic function of NF-κB in MWCNT-induced lung fibrosis. These findings therefore disclose that NF-κB signaling functions as a molecular connection between pro-inflammatory factors and fibrosis development (Dong and Ma, 2019a).

In agreement with the biological functions of fibroblasts and myofibroblasts, copious expression and deposition of fibrotic ECM proteins, such as Collagen I, Collagen III, and fibronectin, are observed in CNT-exposed lungs, especially in fibrotic foci where CNTs deposit and fibroblastic cells accumulate, resulting in pathologic ECM remodeling and tissue scarring (Dong and Ma, 2017a, b). Numerous investigations on this finding have been reported, with traditional methods of collagen analysis, such as Masson’s Trichrome staining and Picro-Sirius Red staining, and antibody-based immunostaining methods that detect specific proteins, such as immunohistochemistry and immunofluorescence, which have been summarized elsewhere (Dong and Ma, 2016b; Vietti et al., 2016). A number of representative studies are listed in Table 1. The accumulation of fibrotic ECM directly indicates the critical role of fibroblasts and myofibroblasts in driving CNT-induced lung fibrosis, and serves as an apparent hallmark of microenvironmental changes induced by CNT exposure.

## Epithelial Cells and Alarmins

Lung epithelial cells are activated as effector cells in response to exposed pathogens and environmental insults. They play important functions in mediating host defense via regulating innate and adaptive immune responses (Holtzman et al., 2014; Leiva-Juarez et al., 2018). Upon exposure to stimuli, injured epithelial cells produce and secrete certain alarmins that can initiate type 2 immune response, such as IL-25, IL-33, and TSLP, demonstrating the critical role of epithelial cells in activating type 2 immunity and tissue fibrosis (Paul and Zhu, 2010; Gieseck et al., 2018; Lloyd and Snelgrove, 2018). A number of studies have shown MWCNTs elevate the levels of IL-25, IL-33, and TSLP in mouse BAL and lung tissues, suggesting the injury and function of epithelial cells induced by MWCNTs in the lung (Wang et al., 2011; Katwa et al., 2012; Beamer et al., 2013; Ronzani et al., 2014; Sager et al., 2014). A few representative studies are listed in Table 1. Moreover, IL-33+ type II pneumocytes (surface epithelial cells of the alveoli) are observed in the vicinity of alveolar macrophages phagocytosing MWCNTs or free MWCNTs, but not in the areas lacking MWCNTs, in the lung by immunohistochemistry assay, directly demonstrating the induced expression of IL-33 in epithelial cells by MWCNTs (Beamer et al., 2013). In cultured C10 mouse epithelial cells, the levels of LDH and IL-33 protein are increased by MWCNTs in a dose-dependent manner, supporting the damage of epithelial cells and the production of IL-33 by these cells stimulated by MWCNTs (Beamer et al., 2013). Importantly, when IL-33 signaling is blocked by using anti-ST2 antibodies or mice with mast cells deficient of ST2, MWCNT-induced type 2 immune response is significantly reduced, compared with control (Katwa et al., 2012; Beamer et al., 2013). These studies reveal that epithelial cells play a crucial role in initiating type 2 immune response via the IL-33/ST2 signaling during CNT-induced lung inflammation and fibrosis. Together, these findings demonstrate that certain type 2 alarmins are induced and trigger the activation of type 2 immune response in CNT-exposed lungs (Figure 1).

Epithelial cells also contribute to the onset of acute inflammation in the lung through producing the alarmin HMGB1 (Andersson and Tracey, 2011; Harris et al., 2012; Magna and Pisetsky, 2014; Huebener et al., 2015). SWCNTs and MWCNTs have been shown to increase the level of HMGB1 in BAL fluid from exposed mice (Jessop and Holian, 2015; Cui et al., 2019). In cultured C10 mouse epithelial cells, it is detected that the production and secretion of HMGB1 are induced by MWCNTs, indicating epithelial cells are a source of HMGB1 in MWCNT-exposed lungs (Jessop and Holian, 2015). By using anti-HMGB1 neutralizing antibodies and Caspase-1 KO mice, HMGB1 is demonstrated to increase IL-1β secretion through NLRP3 inflammasome activation and initiate acute inflammation in MWCNT-exposed mouse lungs (Jessop and Holian, 2015). These findings suggest that HMGB1 functions as a pro-inflammatory alarmin in CNT-exposed lungs and epithelial cells are implicated in CNT-induced acute inflammation through producing pro-inflammatory alarmins (Figure 1).

## Conclusion

Mechanistic understanding of CNT-triggered lung inflammation and fibrosis has identified a variety of immune and structural cells, soluble signaling molecules, and ECM proteins that are evidently induced by CNT exposure and result inmicroenvironmental alterations in the lung. These changes function as pro-inflammatory and/or pro-fibrotic elements that play fundamental roles in promoting the onset and progression of inflammation and fibrosis directly or indirectly, as well as serve as the cellular and molecular links that mediate the interactions between inflammation and fibrosis. The available evidence suggests that the mechanisms and modes of action underlying CNT-induced lung inflammation and fibrosis are in agreement with the overall understanding of inflammation and fibrosis to a great extent, which therefore provides the mechanistic basis for the potential of using CNT-exposed animals as a disease model to study lung inflammation and fibrosis.

## Author Contributions

The author confirms being the sole contributor of this work and has approved it for publication.

## Disclaimer

The findings and conclusions in this report are those of the author and do not necessarily represent the official position of the National Institute for Occupational Safety and Health, Centers for Disease Control and Prevention.

## Conflict of Interest

The authors declare that the research was conducted in the absence of any commercial or financial relationships that could be construed as a potential conflict of interest.

## Abbreviations

α -SMA: α-smooth muscle actin
ARG1: arginase 1
BAL: bronchoalveolar lavage
CCL: chemokine (C-C motif) ligand
CD: cluster of differentiation
CD163: hemoglobin scavenger receptor
CD206: mannose receptor C-type 1 or MRC1
CHIA: chitinase acidic or AMCase
CNT: carbon nanotube
COPD: chronic obstructive pulmonary disease
CXCL: chemokine (C-X-C motif) ligand
ECM: extracellular matrix
Erk: extracellular signal-regulated kinase
FIZZ1: found in inflammatory zone 1 resistin-like molecule α or RELM α or resistin-like- α or RETNL α
GATA-3: GATA-binding protein 3
HMGB1: high mobility group box 1
IL: interleukin
IL-1R: IL-1 receptor
ILC: innate lymphoid cell
iNOS: inducible nitric oxide synthase or NOS2
IPF: idiopathic pulmonary fibrosis
IRF: interferon regulatory factor
Ki-67: marker of proliferation Ki-67
KO: knockout
LDH: lactate dehydrogenase
M1: traditionally activated macrophage
M2: alternatively activated macrophage
MCP-1: monocyte chemotactic protein 1
MHC II: major histocompatibility complex II
MMP: matrix metalloproteinase
MWCNT: multi-walled carbon nanotube
NF- κ B: nuclear factor- κ B
NLRP3: nucleotide-binding oligomerization domain-like receptor: pyrin domain-containing 3
OPN: osteopontin or secreted phosphoprotein 1 or SPP1
PCNA: proliferating cell nuclear antigen
PDGF: platelet-derived growth factor
RANTES: regulated on activation, normal T cell expressed and secreted or CCL5
Smad: Sma- and Mad-related family
ST2: suppression of tumorigenicity 2, receptor for IL-33 or IL-33R, IL-1 receptor-like 1 or IL1RL1 or IL-1 receptor 4 or IL-1R4
STAT: signal transducer and activator of transcription
SWCNT: single-walled carbon nanotube
TGF- β 1: transforming growth factor- β 1
Th: T helper
TIMP1: tissue inhibitor of metalloproteinase 1
TNF- α: tumor necrosis factor-α
TSLP: thymic stromal lymphopoietin
YM1, chitinase 3-like 3 or CHI3L3: or eosinophil chemotactic factor-lymphocyte or ECF-L.
